# Letter to the Editor regarding article “J point elevation in high precordial leads associated with risk of ventricular fibrillation”

**DOI:** 10.1111/anec.12867

**Published:** 2021-07-21

**Authors:** Alper Karakuş, Kadir Uğur Mert, Bülent Görenek

**Affiliations:** ^1^ Department of Cardiology Besni State Hospital Adıyaman Turkey; ^2^ Department of Cardiology Faculty of Medicine Eskisehir Osmangazi University Eskisehir Turkey

Dear Editor,

We have read with great interest the recent article entitled “J point elevation in high precordial leads associated with risk of ventricular fibrillation” by Yuki Hasegawa et al., (2020). In their case–control study, the authors compared the electrocardiogram (ECG) of 35 patients with idiopathic ventricular fibrillation (IVF) versus those of 105 age‐ and gender‐matched control subjects and with those of 15 patients with Brugada syndrome (BrS). They evaluated the frequency of J point elevation ≥0.1 mV in the 3rd and 4th intercostal spaces. The authors concluded that the prevalence of J point elevation in the 3rd intercostal spaces was high in patients with IVF. We appreciate the authors on this successful study and would like to address some points to merit more attention.

There are conflicting results of population‐based studies of J point elevation seen on surface ECG. The location of the J wave also varied in the different patient groups (Rosso et al., 2008). Importantly, this pattern can be seen in the setting of younger age, lower heart rate, electrolyte imbalance, hyperthermia (fever), and usage of pharmacological agents (antiarrhythmics, antianginals, psychotropics, or anesthetics/analgesics drugs) (Antzelevitch et al., 2017; Nam, 2012). Furthermore, the presence of the abovementioned factors in J‐wave syndrome during the peri‐event period can trigger the malignant arrhythmia and influence the association with sudden cardiac arrest (Priori et al., 2015). Therefore, it is necessary to include clinical data of modular factors on analysis for making clear the relationship between J point elevation in high precordial leads and ventricular fibrillation episodes. Additionally, the statistical measurement used in the study seems limited in its ability to assess relative risk. In addition, there are some methodological issues and scant data regarding skewness in the article. Especially, small sample sized make us think can be a violation of normality and so the Mann‐Whitney U test could have been appropriate. Also, comparisons between IVF patients with and without J point and BrS should have been used one‐way ANOVA or Kruskal‐Wallis test because there are three groups (Armitage, 2008).

Conclusively,

It can be argued that J point elevation in the 3rd intercostal spaces may be a marker for “increased risk” in idiopathic ventricular fibrillation. The results could only reflect the ECG correlates of IVF but not its prognostic power for ventricular fibrillation episodes. It would be safe to say the link between J point elevation in high precordial leads and malignant arrhythmias after evaluation of the relation between the abovementioned clinical variables and ECG which was assessed by using Cox proportional hazards regression analysis in this study.

## CONFLICT OF INTEREST

The authors declare that they have no known competing financial interests or personal relationships that could have appeared to influence the work reported in this paper.

## AUTHOR CONTRIBUTIONS

A.K., K.U.M., and B.G. involved in concept, design, supervision, analysis and interpretation, literature search, writing, and critical review.

## ETHICAL APPROVAL

The protocol was approved by the Institutional Ethics Committee, Eskisehir Osmangazi University, Turkey.

## 1 DATA AVAILABILITY STATEMENT

Data openly available in a public repository that issues datasets with DOIs.
